# Using mechanism similarity to understand enzyme evolution

**DOI:** 10.1007/s12551-022-01022-9

**Published:** 2022-12-03

**Authors:** António J. M. Ribeiro, Ioannis G. Riziotis, Jonathan D. Tyzack, Neera Borkakoti, Janet M. Thornton

**Affiliations:** European Bioinformatics Institute - European Molecular Biology Laboratory, Wellcome Trust Genome Campus, Hinxton, Cambridge, CB10 1SD UK

**Keywords:** Enzyme mechanism, Enzyme evolution, Catalytic steps

## Abstract

Enzyme reactions take place in the active site through a series of catalytic steps, which are collectively termed the enzyme mechanism. The catalytic step is thereby the individual unit to consider for the purposes of building new enzyme mechanisms — i.e. through the mix and match of individual catalytic steps, new enzyme mechanisms and reactions can be conceived. In the case of natural evolution, it has been shown that new enzyme functions have emerged through the tweaking of existing mechanisms by the addition, removal, or modification of some catalytic steps, while maintaining other steps of the mechanism intact. Recently, we have extracted and codified the information on the catalytic steps of hundreds of enzymes in a machine-readable way, with the aim of automating this kind of evolutionary analysis. In this paper, we illustrate how these data, which we called the “rules of enzyme catalysis”, can be used to identify similar catalytic steps across enzymes that differ in their overall function and/or structural folds. A discussion on a set of three enzymes that share part of their mechanism is used as an exemplar to illustrate how this approach can reveal divergent and convergent evolution of enzymes at the mechanistic level.

**Supplementary Information:**

The online version contains supplementary material available at 10.1007/s12551-022-01022-9.

## Introduction

Enzymes are crucial and abundant components of living organisms (The UniProt Consortium 2021) and are increasingly important in industrial settings (Chapman et al. 2018). How enzymes have evolved completely new functions is still an open question in biology. The understanding of the basic principles of enzyme evolution would pave the way for the design of new mechanisms from scratch. Past studies on the evolution of enzymes have looked at the sequence, structure, and reactions of enzymes as well as the relationships between these variables (Galperin et al. 1998; Gerlt and Babbitt 2001; Gherardini et al. 2007; Furnham et al. 2012; Martínez Cuesta et al. 2015; Baier et al. 2016). Some main observations from these studies include (a) changing the specificity of the substrate is a relatively common event, when compared to change in chemical function; (b) changes in chemical function are frequent enough and happen between all EC classes; (c) most variation in enzyme function comes from divergent evolution, with a few structural folds accounting for the majority of known enzymatic reactions; (d) convergent evolution of new enzyme functions is relatively common at the reaction level, since it is typical for EC sub subclasses to be associated with more than one three-dimensional fold.

While the above investigations are insightful, and can be done across most of the enzymatic space, due to the availability of sequence (The UniProt Consortium 2021), structural (Berman et al. 2003; wwPDB consortium 2019; Armstrong et al. 2020; Andreeva et al. 2020; Sillitoe et al. 2021), and reaction (Kanehisa and Goto 2000; Fleischmann et al. 2004; Bansal et al. 2022) databases, the ideal scenario would be to include mechanistic data in the analysis. The enzyme mechanism, defined here as the sequence of chemical steps undertaken by the enzyme in each catalytic cycle, is the most detailed description of how individual enzymes work. While it is possible to study the correlation between the enzyme sequence (or structure) and the enzyme reaction by comparing and mapping the changes between one and the other, true understanding, as well as the capacity to make predictions and design changes, only comes when the underlying mechanisms and links of causation are established.

Studying enzyme evolution at this mechanistic level is challenging. Determining the mechanism of individual enzymes involves laborious experimental and computational studies and can lead to ambiguous results for some catalytic steps. In addition, the data produced are complex, involving the description of several chemical species (reactants, cofactors, and catalytic residues) which are modified along multiple chemical steps in a three-dimensional setting (the active site). Finally, the reporting of enzyme mechanisms in the literature is not standardised, making the direct comparison of enzyme mechanism proposals complex, especially while comparing proteins of different families.

## The M-CSA and the rules of enzyme catalysis

To overcome these difficulties, for many years, our group has been searching the literature for data on enzyme mechanisms and catalytic sites and manually curating this knowledge in a standardised way (Holliday et al. 2005; Furnham et al. 2013). This effort has resulted in the current version of the M-CSA database (Mechanism and Catalytic Site Atlas) (Ribeiro et al. 2018), freely available at www.ebi.ac.uk/thornton-srv/m-csa/, containing the detailed description of the reaction mechanisms of 734 enzymes. This dataset has been used in the past, both by us (Ribeiro et al. 2020a, b) and others (Stourac et al. 2019; Andersen et al. 2021; Miller et al. 2019), to study some aspects of the function and evolution of enzymes.

Of particular relevance to the present mini review, we highlight a study (Bartlett et al. 2003) made on 27 pairs of divergent enzymes showing that new overall reactions can be created by changing some catalytic steps, while conserving others. Since the description of each catalytic step contains information of the active site (including catalytic residues, cofactors, and reactants) and the occurring chemistry, this analysis provides a fundamental explanation of how the mutation of catalytic residues or other changes in the active site led to the change of each catalytic step, and, in turn, of the overall enzyme function. More recently, we have been working on reproducing this powerful analysis in an automated way and across all the available enzyme families. Furthermore, we would also like to extend the analysis to convergent evolution scenarios.

Our last effort in this regard (Ribeiro et al. 2022) has been the construction of a machine-readable dataset of all the chemistry observed in the M-CSA database, at the catalytic step level. This data, created by parsing all the chemical diagrams of the individual catalytic steps in the database, was then distilled into a set of catalytic rules which describe only the reactive part of the molecules involved in bond changes. In the current version of the rules, this is defined as the reaction centres (atoms directly involved in the bond changes) plus atoms up to two bonds away. Furthermore, carbon or hydrogen atoms that are exactly two bonds away from the reaction centres are deemed undistinguishable (see Fig. 2B for an example). These rules, codified and stored in the database as SMARTS reaction queries, allow for the comparison of catalytic steps, and the identification of common patterns of catalysis across enzymes, independently of their sequence or structural conservation or overall reaction similarity. In the rules page of the M-CSA website — www.ebi.ac.uk/thornton-srv/m-csa/rules/ — it is possible to browse all the catalytic rules ordered by their rate of occurrence in different mechanisms and enzymes.

## Overview of the shared chemical steps across enzymes with different functions and folds

Of the 1853 rules that are observed in all catalytic steps annotated in the M-CSA (forward and reverse directions of the same rule are analysed as one), a majority of 1562 are seen in just one enzyme. Although the exact figures are dependent on the definition used to create the rules (and we continue to test new definitions in order to make rules that are more general), this number indicates that most enzyme mechanisms contain some unique chemistry. We can identify 417 rules that are observed in more than one catalytic step. In other words, there are at least 417 groups of catalytic steps that perform the same chemistry. Of these groups, 126 contain only catalytic steps belonging to the same enzyme. The remaining 291 rules, which will be the focus of the following analysis, are observed in more than one enzyme (these rules and associated enzymes can be browsed at www.ebi.ac.uk/thornton-srv/m-csa/rules/).

**Fig. 1:**
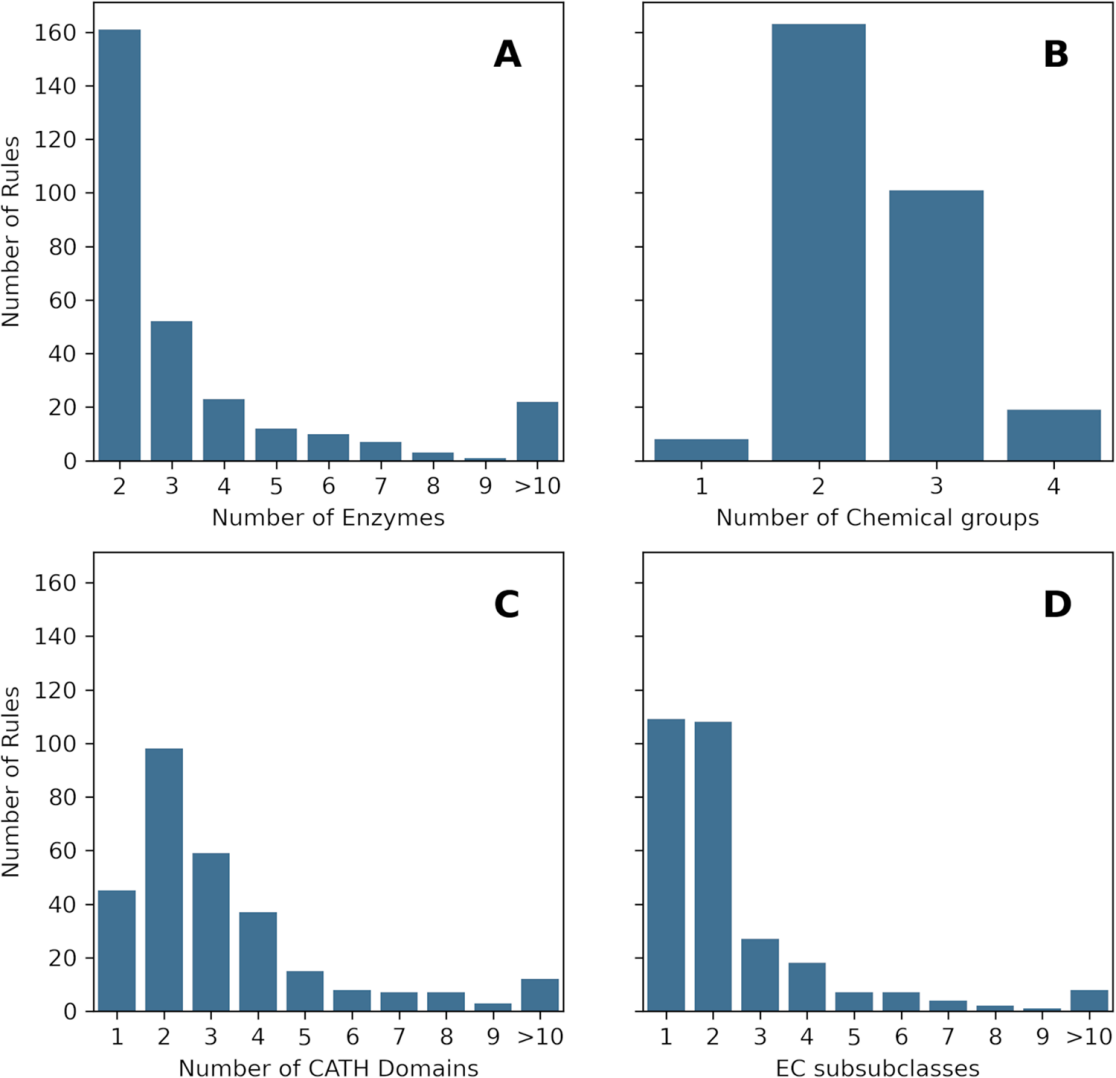
Distribution of the 291 catalytic rules that are observed in more than one enzyme across different variables. **A** Distribution of the shared rules by the number of enzymes they appear in. **B** Distribution of the shared rules by the number of molecules involved in the rule (whatever number is larger on either side of the reaction). **C** Distribution of the shared rules by the number of CATH superfamilies they are observed on. **D** Distribution of the shared rules by the number of EC sub subclasses (the third level of the EC classification)

Figure 1 shows the distribution of non-unique rules according to several parameters. Most of these rules (161) appear in just two enzyme mechanisms, and a progressively smaller number of them is shared by three or more enzymes (Fig. 1A). There are 22 rules that are observed in 10 or more enzymes. The distribution is similar for the number of steps the rules appear on, with a slight skew towards the right, since some of the rules appear in more than one catalytic step of the same enzyme (Fig. SI-1). Shared rules typically involve two or three chemical groups (Fig. 1B), in a distribution that is roughly the same as the one for the unique rules (Fig. SI-2). The distribution of CATH domains is shown in Fig. 1C. There are 45 shared rules that are observed in a single CATH domain, which might indicate that each of these are evolutionarily conserved across the enzymes where they are observed. A more detailed analysis is required to confirm this hypothesis, since some of these rules might be trivial (such as the protonation of a single residue by water) or they may be performed by different catalytic residues (and in that case they are examples of convergent evolution). Conversely, rules that are associated with more than one CATH superfamily might refer to chemistry that emerged independently. Some rules, like rule “a”, discussed below in the test case, present both divergent and convergent origins, for different enzymes where they are observed. Finally, Fig. 1D shows that a significant portion of the shared rules (109) are observed in enzymes that catalyse reactions belonging to the same EC sub subclass (third level of the EC classification), while the remaining 182 are associated with at least two EC sub subclasses. The lack of a clear correlation between the chemical steps and the EC classification shows the necessity of looking at mechanisms to thoroughly understand the evolution of enzyme function since enzymes might catalyse the same reaction using different catalytic steps or catalyse different reactions with shared catalytic steps and similar mechanisms.

## Test case — using the catalytic rules to find similar catalytic steps in related and unrelated enzymes

In previous studies focused on the evolutionary trajectory of enzymes, cases of divergent evolution have been identified by searching for enzymes that have similar sequence or structural fold but catalyse different reactions. Convergent evolution, on the other hand, has been identified among enzymes that catalyse the same chemical reaction but have unrelated sequences and folds. Here, we show how another enzyme property, mechanism similarity, can be used to identify both evolutionary scenarios.

### Three enzymes with a common catalytic step

**Fig. 2:**
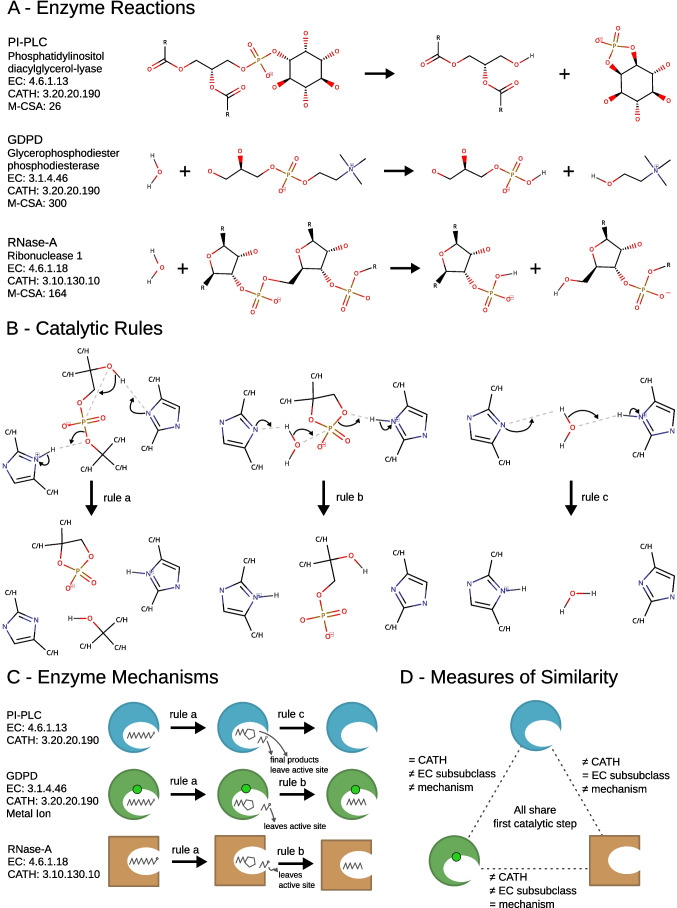
Reactions, mechanisms, and structural and chemical classifications of three enzymes that share one catalytic step, PI-PLC, GDPD, and RNase-A. **A** The balanced chemical reactions each enzyme catalyses. **B** The catalytic rules that are observed in the three mechanisms. **C** Schematic representation of the mechanisms of each enzyme. The shapes of the enzymes represent their catalytic folds, i.e. — RNase-A has a different fold. A metal ion in GDPD is represented by a green circle. **D** Scheme showing the differences and similarities between pairs of the three enzymes

To illustrate this concept, we selected a catalytic rule that is observed in three enzymes, whose similarities have been previously remarked (Hondal et al. 1998; Shi et al. 2008). Two of these enzymes belong to the same CATH superfamily whereas a different pair shares the same EC sub subclass. All three enzymes have simple two-step mechanisms, where the first step is common to all mechanisms. Figure 2 shows the three reactions catalysed by each of these enzymes (panel A), all the catalytic rules defined in their mechanisms (panel B), and a schematic representation of the three mechanisms (panel C). The rule shared by the three enzymes is identified in the figure as rule “a”.

The first enzyme shown in Fig. 2A, phosphatidylinositol diacylglycerol-lyase (PI-PLC, EC: 4.6.1.13), catalyses the breaking of a P-O bond leading to the formation of a cyclic phosphate inositol (Essen et al. 1997; Hondal et al. 1998; Ryan et al. 2001). The second enzyme, glycerophosphodiester phosphodiesterase (GDPD, EC: 3.1.4.46), hydrolyses a P-O bond in a similar molecular environment (a 2-ammonioethyl group is pictured, but the enzyme will work with other R groups) (Shi et al. 2008). Lastly, ribonuclease A (RNase-A, EC: 4.6.1.18) hydrolyses a P-O bond between two RNA bases (Raines 1998; Gu et al. 2013). Remarkably, the second and third reactions are quite similar, despite belonging to different EC classes, which highlights the difficulties of using a hierarchical tree-like structure (as used in the EC nomenclature) to categorise the multidimensional chemical space.

Figure 2B shows the three rules observed in the three mechanisms. All the rules use a pair of His residues as proton acceptors/donors. In rule “a”, an intramolecular nucleophilic substitution leads to the formation of a cyclic phosphate and a separate leaving group. Rule “b” describes the opening of a cyclic phosphate with the help of a water molecule and the His residues. Rule “c” is simply the exchange of protons between His residues with the help of a water molecule.

Figure 2C clarifies how each enzyme operates. All the three enzymes follow rule “a” as the first catalytic step, which results in the formation of a cyclic phosphate and the leaving group as another product. For PI-PLC, these are the final products of the reaction, and the second catalytic step is simply the regeneration of the active site His residues, according to rule “c”. The other two enzymes follow another catalytic step, rule “b”, to open the cyclic phosphate, which requires a water molecule.

### Evolutionary relationships and active site comparison

The three enzymes described above all share the first catalytic step of their mechanisms, but with respect to other measures of similarity, this group is not so homogenous (see Fig. 2D). PI-PLC and GDPD belong to the same CATH superfamily (CATH: 3.20.20.190), denoting a divergent evolutionary relationship, while RNase-A has a completely different fold (CATH: 3.10.130.10), which means its function arose independently from the others. At the EC level, PI-PLC and RNase-A share the same sub subclass, while GDPD belongs to a different EC class altogether. When it comes to the mechanism (which correlates to the reaction similarity), GDPD and RNase-A follow the same exact mechanism, as opposed to PI-PLC.

This example paints an intricate picture about some of the possibilities in enzyme evolution. Enzymes may diverge to catalyse different overall reactions, even if using some common catalytic steps (PI-PLC and GDPD). Two unrelated enzymes may converge to catalyse very similar reactions using an identical mechanism (GDPD and RNase-A). Finally, two unrelated enzymes might be catalysing different reactions using partially the same mechanism (PI-PLC and RNase-A).

**Fig. 3:**
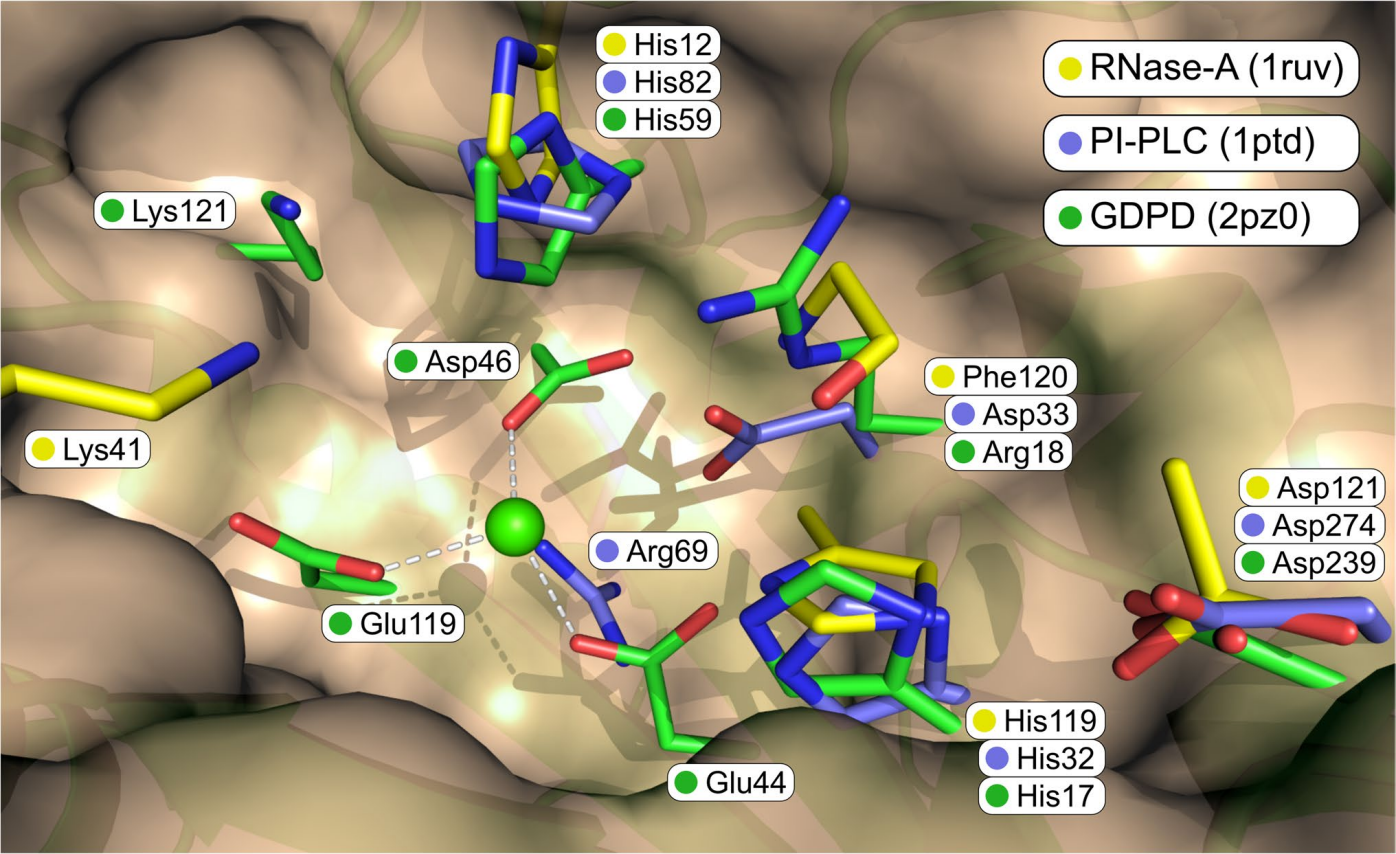
Superposition of the three-dimensional structure of the active sites of PI-PLC (purple atoms), GDPD (green), and RNase-A (yellow). Only side chain atoms are shown for all residues, except for Phe120, for which only main chain atoms are represented. The green sphere represents the magnesium metal ion in GDPD. Picture created with Pymol (Schrödinger 2015)

The analysis of the full list of catalytic residues of each enzyme and the superposition of the three active sites (Fig. 3) reveal further complexity (Heinz et al. 1995; Ladner et al. 1997; Shi et al. 2008). Firstly, it is remarkable that the active site of the evolutionarily unrelated enzyme (RNase-A, with carbon atoms in yellow) superimposes almost perfectly with the active sites of the unrelated enzymes. For the three catalytic residues that are conserved in the three enzymes (His12/82/59, His119/32/17, and Asp121/274/239), the side chains are positioned in roughly the same place, while the main chains, as expected, since the fold is completely different, are not. Secondly, for the two related enzymes (PI-PLC and GDPD), except for the three residues already mentioned, there are no catalytic residues in common. Surprisingly, one of these enzymes (GDPD) contains a magnesium metal ion in the active site, coordinated by three Glu residues, while the other (PI-PLC) presents an Arg residue, in the same position. These positively charged metal and residue have the same role of stabilising the negatively charged phosphate and transition state. Thirdly, both GDPD and RNase-A feature a Lys residue originating from different parts of the backbone but pointing in the same general direction. And finally, the mainchain nitrogen of Phe120 in RNase-A, annotated specifically in the database as having a role in the stabilisation of the negatively charged transition states, superimposes well with Arg18, which has the same role in GDPD.

In summary, the three catalytic residues directly related with the bond changes are conserved (or evolved independently) in the three active sites, but the stabilisation of the negatively charged transition state is achieved by three different methods. PI-PLC uses the positively charged Arg69, GDPD contains a metal ion in the active site together with Lys121 and Arg18, and RNase-A uses Lys41 and the nitrogen main chain of Phe120.

## Conclusions

Enzymes are known to catalyse at least 15,000 reactions (Bansal et al. 2022) using a limited toolset of catalytic residues and cofactors. It is to be expected that some chemical patterns will be observed in more than one enzyme. These similarities at the mechanism and catalytic step level can be attributed to divergent evolution and the conservation of catalytic residues or to have emerged independently through convergent evolution. By analysing a set of rules of enzyme catalysis derived from the mechanisms of 734 enzymes annotated in the M-CSA database, we found that among 1853 rules, 291 were found in more than one enzyme. Shared rules are observed in enzymes with the same or different CATH superfamilies, denoting common or distinct ancestry, respectively, and they are observed in enzymes that have the same and different EC sub subclasses, a proxy for chemical similarity.

We then used a set of three enzymes that share a common catalytic rule to better illustrate this diversity. The analysis of the chemical reactions, mechanisms, and active sites of these enzymes showed how the same chemical step might be catalysed in enzymes with different folds, and how changing just one catalytic step might lead to different functions. Interestingly, these enzymes have an almost identical set of three catalytic residues that are directly involved in the formation and breaking of bonds while, at the same time, different active site residues and configurations when it comes to the stabilisation of the transition states.

The overview of the distribution of the shared rules across different CATH and EC codes, as well as the presented test case, shows the complexity and diversity of enzyme evolution. Ongoing research is being focused towards answering more detailed questions. How do mutations lead to the change of function, after gene duplication? How do the changes of function fit the phylogenetic trees? Do the same changes of function arise multiple times in different organisms? How common is convergent evolution? Do convergent enzymes that catalyse the same reaction typically use a common active site and mechanism? Our unique dataset of enzyme mechanisms should enable us to answer these and other questions at the mechanistic level, which has been done before only for a selected number of cases. Future improvements in our analysis that will be required to tackle this problem include the need to take the whole mechanism similarity into account, instead of focusing solely in a single catalytic step at a time, and the inclusion of homology data, multisequence alignments, and phylogenetic trees to better establish the evolutionary relationships.

## Supplementary Information

Below is the link to the electronic supplementary material.

Supplementary file1 (PNG 36 KB)

Supplementary file2 (PNG 43 KB)
