# Supplementary figures and images for: Using mechanism similarity to understand enzyme evolution
Source: Biophys Rev. 2022 Dec 3;14(6):1273–80. doi: 10.1007/s12551-022-01022-9 (PMC9842563; doi:10.1007/s12551-022-01022-9)

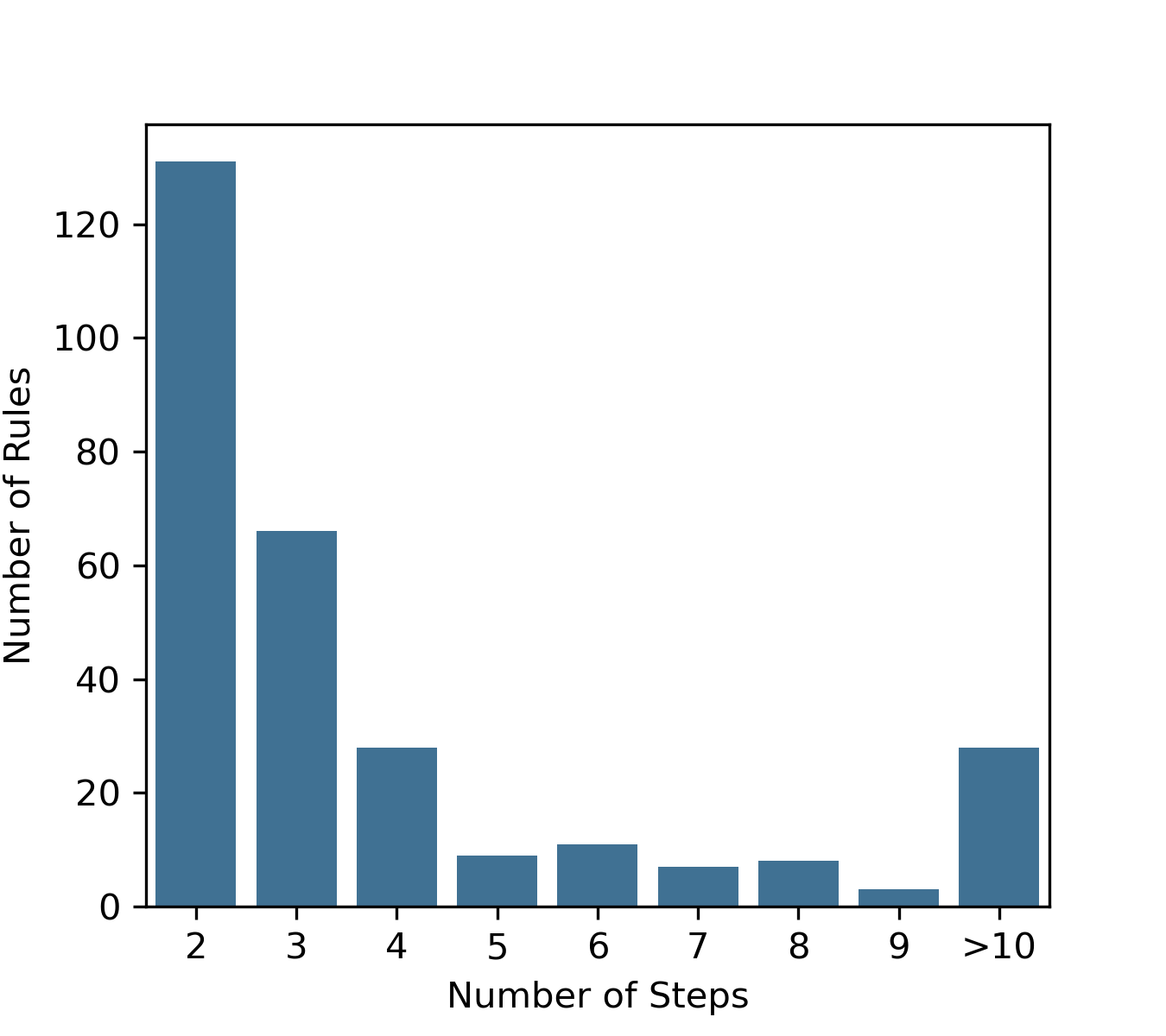

Supplement: Supplementary file 1 — Supplementary file1 (PNG 36 KB) [file 12551_2022_1022_MOESM1_ESM.png]

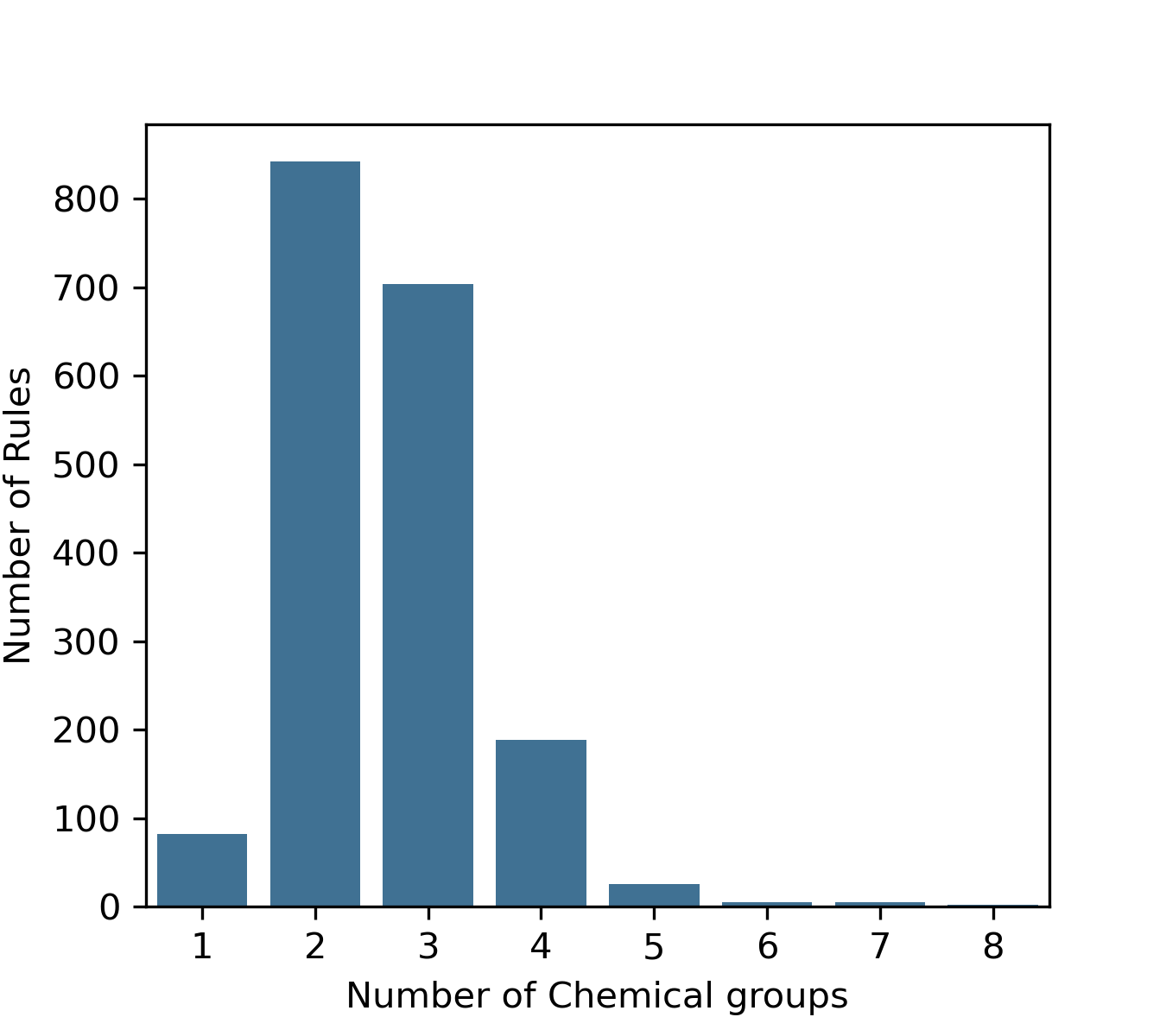

Supplement: Supplementary file 2 — Supplementary file2 (PNG 43 KB) [file 12551_2022_1022_MOESM2_ESM.png]
